# Health-Related Financial Catastrophe, Inequality and Chronic Illness in Bangladesh

**DOI:** 10.1371/journal.pone.0056873

**Published:** 2013-02-25

**Authors:** Md. Mizanur Rahman, Stuart Gilmour, Eiko Saito, Papia Sultana, Kenji Shibuya

**Affiliations:** 1 Department of Global Health Policy, The University of Tokyo, Tokyo, Japan; 2 Department of Population Science and Human Resource Development, University of Rajshahi, Rajshahi, Bangladesh; 3 Department of Statistics, University of Rajshahi, Rajshahi, Bangladesh; Erasmus University Rotterdam, The Netherlands

## Abstract

**Background:**

Bangladesh has a high proportion of households incurring catastrophic health expenditure, and very limited risk sharing mechanisms. Identifying determinants of out-of-pocket (OOP) payments and catastrophic health expenditure may reveal opportunities to reduce costs and protect households from financial risk.

**Objective:**

This study investigates the determinants of high healthcare expenditure and healthcare- related financial catastrophe.

**Methods:**

A cross-sectional household survey was conducted in Rajshahi city, Bangladesh, in 2011. Catastrophic health expenditure was estimated separately based on capacity to pay and proportion of non-food expenditure. Determinants of OOP payments and financial catastrophe were estimated using double hurdle and Poisson regression models respectively.

**Results:**

On average households spent 11% of their total budgets on health, half the residents spent 7% of the monthly per capita consumption expenditure for one illness, and nearly 9% of households faced financial catastrophe. The poorest households spent less on health but had a four times higher risk of catastrophe than the richest households. The risk of financial catastrophe and the level of OOP payments were higher for users of inpatient, outpatient public and private facilities respectively compared to using self-medication or traditional healers. Other determinants of OOP payments and catastrophic expenses were economic status, presence of chronic illness in the household, and illness among children and adults.

**Conclusion:**

Households that received inpatient or outpatient private care experienced the highest burden of health expenditure. The poorest members of the community also face large, often catastrophic expenses. Chronic illness management is crucial to reducing the total burden of disease in a household and its associated increased risk of level of OOP payments and catastrophic expenses. Households can only be protected from these situations by reducing the health system's dependency on OOP payments and providing more financial risk protection.

## Introduction

The fundamental role of a healthcare system is not only to improve population health but also to protect households from financial catastrophe associated with illness [1]. Out-of-pocket (OOP) payments for healthcare can cause households to incur catastrophic expenditures, pushing them into poverty [2], [3]. Globally, approximately 44 million households face catastrophic health expenditure annually, and about 25 million households are pushed into poverty by their health expenses [4], [5]. In countries where risk pooling mechanisms are available people are protected from catastrophic spending [4] but many low- and middle-income countries experience high OOP payments and lack risk-sharing mechanisms, forcing households into hardship, asset depletion, debt, reduction of essential consumption, and sometimes financial catastrophe [4]–[11].

Like many developing countries, Bangladesh is also facing the double burden of disease [12], [13], OOP payments remain the most important source of funding for healthcare, and health insurance is almost nonexistent except for small pockets of NGO-financed schemes [14]. Despite the possibly high incidence of catastrophic expenses and high OOP expenditure in Bangladesh [15], [16], we are not aware of any study detailing determinants of OOP payments and catastrophic expenditures. To date, the only two studies reporting overall incidence of catastrophic expenses in Bangladesh are multi-country studies that present contradictory findings because of data limitations and methodological differences [16], [17]: one of them found a very low (1.2%) incidence of catastrophic expenditure [17], while the other found a very high incidence (15%) [16]. They also did not explore variations in OOP payments and catastrophic spending by healthcare facility, or by household or individual characteristics such as the presence of chronic illness. Several studies suggested that illness among a child or adult, presence of chronic illness [18]–[21], lack of health insurance [9], [10], [17], [22], and use of inpatient or outpatient care [8], [10], [19], [21]–[25] are key factors in high OOP payments and catastrophic health expenditure, but very few studies have considered these factors simultaneously. An interesting study in India examined determinants of OOP payments but did not extend the analysis to the associated problem of catastrophic expenditure [21]. Our study expands on this methodology to include assessment of the incidence of catastrophic expenditure, which is a key measure of the extent of financial risk protection as it judges whether the existing health financing system is able to protect its residents from the consequences of OOP payments [26].

Thus, previous estimates may have provided an incomplete picture of the impact of medical expenses on populations with a high prevalence of chronic illness and limited risk-pooling mechanisms. In designing healthcare financing systems, policy makers need to understand determinants not just of OOP payments, but also the related problem of catastrophic health expenditure associated with high OOP payments [17]. To address these questions, the study aims to investigate two closely linked phenomena: the determinants of OOP payments and catastrophic expenditure in Bangladesh. These analyses are conducted using double hurdle and Poisson regression models in combination with a probability survey.

## Data and Methods

### Study area

The study was conducted in Rajshahi city, Bangladesh, which is the third largest city in the country and broadly representative of many urban areas in Bangladesh [27]. Rajshahi has a population of 4.59 million, with an average household size of about five. The literacy rate is 71% and 62% for males and females respectively [27]. In rural areas of Bangladesh, there are some supplementary health financing programs such as demand-side financing (DSF), which reduce financial barriers to maternal healthcare among poor women. However, these programs do not exist in urban areas [28], where households suffer more illness, particularly non-communicable diseases (NCDs), and use more health facilities compared to rural households. Therefore, this study focused on urban areas of Rajshahi, in order to examine how urban households deal with OOP health expenditure in the absence of risk protection mechanisms like DSF or health insurance.

### Study design

A cross-sectional three-stage cluster sampled household survey was performed during August to November 2011. The primary sampling unit (PSU) was the *Mahallah*, the lowest administrative region of a Bangladeshi city. In the first stage, 40 clusters were randomly selected from 159 eligible PSUs with probability proportional to size. In the second stage, a fixed number (40) of buildings was selected by systematic random sampling from each chosen cluster based on a household listing operation to provide the necessary frame for selecting buildings. During the final stage, one household was randomly selected from each building, with a target sample of 1600 households. Of these households, only seven refused interview or were not available to be interviewed, and the final effective sample size was 1593 households, resulting in a response rate of 99.6%.

### Data collection

Respondents were administered a structured questionnaire developed based on the Bangladesh Household Income and Expenditure Survey (HIES) [29], and the Living Standards Measurement Survey (LSMS) [30]. The questionnaire was translated from English to Bengali and a pilot study was performed in order to detect implementation difficulties. Field activities were supervised by the study coordinator with the help of the University of Rajshahi, Bangladesh. Twenty-seven interviewers (social science, demography and statistics graduates with experience in survey methods) and five supervisors were recruited to administer this survey. All of them received 10 days' training and two days of practical sessions on the content of the questionnaire, techniques to elicit more information and strategies for obtaining complete and reliable data. For clarification of the research purpose, an interviewer and supervisor operational manual was provided two days before their training started, to ensure they understood their duties and responsibilities.

The respondents in this study were the women in the household, the household head, or the most knowledgeable person in the household, where necessary. Informed consent was obtained prior to conducting the interviews. The survey questionnaire contained two main sections: the household questionnaire and the individual illness questionnaire. The household questionnaire covered survey and household identification and household consumption expenditure, including food consumption, non-food expenditure, housing and durable goods; the individual questionnaire contained demographic information such as age, sex, marital status, education and occupation of individuals in the household, and health problems in the past 30 days. The food consumption section covered purchased, home-produced and in-kind consumption in the past 30 days or past 12 months prior to interview. Following the same recall process as food consumption, the non-food expenditure section also covered purchased and in-kind goods. Housing rent or equivalent rents were recorded in the past 30 days and the durable goods section recorded detailed information on number of items, duration, present and past value of the most recent items in the one year recall period. Cost of medical expenses including fees (consultation/investigation fees, blood tests, etc.), drugs and medical supplies, transport costs for patients and accompanying family members, and other costs were recorded for each episode of illness in the past 30 days prior to interview. All expenditure was recorded in the Bangladeshi currency, taka (TK). In addition, data on timing and cost of all episodes of illness, care-seeking behavior and inpatient or outpatient care were recorded for the past 30 days prior to interview.

### Measures of the burden of OOP payments

Consistent with common definitions, OOP healthcare expenditure was defined as ‘catastrophic’ if it exceeded 40% of household non-food expenditure or capacity to pay in the past 30 days [5], [17], [31], [32]. Total household consumption expenditure was calculated according to the living standard measurement survey guidelines [33] and household consumption quintile was determined using the approach of Xu and colleagues [17]. Household consumption expenditure is the sum of food consumption, non-food expenditure, housing, and durable goods. Catastrophic healthcare expenditure and consumption quintile were calculated using household total consumption expenditure, capacity to pay and equivalent household size. This equivalent scale is used, rather than actual household size, because in low-and middle-income countries household consumption expenditure increases with increases in household size but that increase is less than proportionate to the increase in household size [17]. We also calculated the ratio of medical expenses intensity proposed by Dror and colleagues [21]. This medical expenses intensity ratio was estimated by dividing the average medical expenses per episode of illness by the average consumption expenditure per household member. We excluded from this analysis the households whose household members did not suffer any kind of illness in the recall period. This ratio was then calculated for each expenditure quintile, as a measure of the burden of OOP payments standardized for illness intensity and household size.

### Statistical analysis

Descriptive statistics were calculated using the mean (confidence interval), median (inter-quartile range) or frequency and proportions as appropriate. Trend tests were performed using the Mantel-Haenszel chi-square test for categorical variables and linear regression analysis for continuous variables, with ordinal numbers (1–5) assigned to the quintile categories. Double hurdle and Poisson regression models were used to identify the determinants of OOP payments and catastrophic expenditure, respectively. A brief description and motivation of the models is given below.

### *The double hurdle regression model*

Reporting of zero expenditure is quite common in household consumption expenditure surveys. For example, both medical and tobacco consumption expenditure are zero for many individuals or households over a survey recall period. In addition, participation in expenditure and the magnitude of expenditure may not be statistically independent [34], [35], and the same stochastic process may not affect participation and consumption level decisions. We used a double hurdle model to overcome these problems [23], [35], [36]. This model requires a subject to pass a consumption decision hurdle before the level of consumption can be modeled. The first hurdle involves the decision about whether or not to participate in healthcare consumption (the participation decision, modeled in the double hurdle model with a probit function). It is reasonable to assume that participation in healthcare spending is influenced by social and demographic factors [35], [37]. The second hurdle concerns the level of health expenditure (the consumption decision, handled with a Tobit function). Thus the model uses information on both the probability and magnitude of expenditure simultaneously in assessing predictors of consumption.

The double hurdle model was used to assess the relationship between demographic and household variables and the size of OOP expenses. The dependent variable for the probit model is a dichotomous variable that indicates whether OOP expenses were incurred (the participation decision). The Tobit regression model analyses the natural logarithm of OOP payments as a function of the covariates (the consumption decision). This model can be presented symbolically through two related equations for participation and consumption.

Observed consumption:
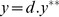
(1)Participation equation:
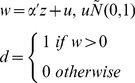
(2)Consumption equation:
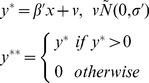
(3)Where d is a latent variable describing the household's decision to participate in the OOP healthcare expenditure, y^*^ is another latent variable describing household level of healthcare expenditure, y is the observed dependent variable (household expenditure on healthcare expenditure), z is a vector of variables explaining the participation decision, and x is a vector of variables explaining the expenditure decision. According to Jones, the likelihood function can be written as [34]:
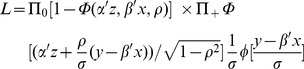
(4)Where zero consumption is denoted as 0 and positive consumption is indicated with a +. In this likelihood function, *p* then denotes probability of expenditure, 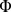 and 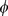 denote distributions and density functions, respectively, and 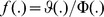. The coefficients for the model are then obtained by maximizing the likelihood (equation (4)).

### Poisson regression model

In the case of rare events, Poisson regression can provide more accurate estimates than logistic regression [38], [39]. Because catastrophic health expenditure can be a rare event, in our study a multiple Poisson regression model was used to identify the determinants of catastrophic expenditure, with model selection based on backward stepwise model-building. This model is well-established for the analysis of counts of rare events [38], [39].

All analyses at both the univariate and multiple regression stages were adjusted for the probability sample design. Statistical analysis was performed using Stata/SE Version 12.0.

### Covariates

The study modeled households' OOP health payments and risk of catastrophic expenditure as a function of household characteristics and economic status and presence of illness and care-seeking behavior, using average illness per child and adult as a measure of illness [20]. Past studies suggested that average number of illnesses per child and adult is less likely to incur bias due to household age structure and more accurately reflects disease occurrence within a household than absolute number of illnesses [20]. In Bangladesh, households often use local, privately-run traditional healers or pharmacists as their prime point of care, and health-seeking behavior in this study was thus classified in three forms: traditional healers/self-medication/no care, outpatient, or inpatient services. Outpatient and inpatient services could be public or private facilities, but traditional healers, pharmacists and other forms of unregulated care provider are always privately run. The small number of respondents receiving inpatient care in this sample precluded separate presentation of this variable by private and public type, but outpatient facilities were divided into public and private facilities. We also could not consider the role of NGO providers separately, because very few households (13 households) in the study sample used NGO-based health services. As a result, these services were combined with private outpatient services during analysis. In our study, care-seeking behavior was then grouped into five categories: inpatient care included those staying overnight in either a hospital or clinic; outpatient public facilities included district/sadar hospitals, maternal and child welfare centers (MCWC), urban health centers, family welfare centers (FWC), government satellite clinics, diabetic centers, other government facilities; outpatient private facilities included private hospitals or clinics, NGO clinics or satellite clinics, and qualified allopathic practitioners (MBBS doctors); both outpatient public and private included those who used outpatient public and private services simultaneously in the past 30 days, self-medication including drugs obtained at a pharmacy or drugstore, kabiraj or spiritual healers, homeopathic practitioners, shops, other traditional healers, or no service of any kind.

### Ethical considerations

This study received ethical approval from the Ethics Committee of The University of Tokyo and the Bangladesh National Research Ethics Committee, with reference number BMRC/NREC/2010-2013/1161. About one third of the population in Rajshahi city are still illiterate and even written consent is not common practice among them. Therefore, a consent form to obtain and document verbal or written consent from respondents was proposed and approved by the Ethics Committee together with the study protocol. Prior to the interview, our enumerator carefully read the consent form to the subject and then very briefly explained the aims and importance of the study. This consent form contained information on the objectives of the study, risks, benefits and freedom of participation, and confidentiality.

## Results

### Background characteristics and OOP payments

**Figure 1:**
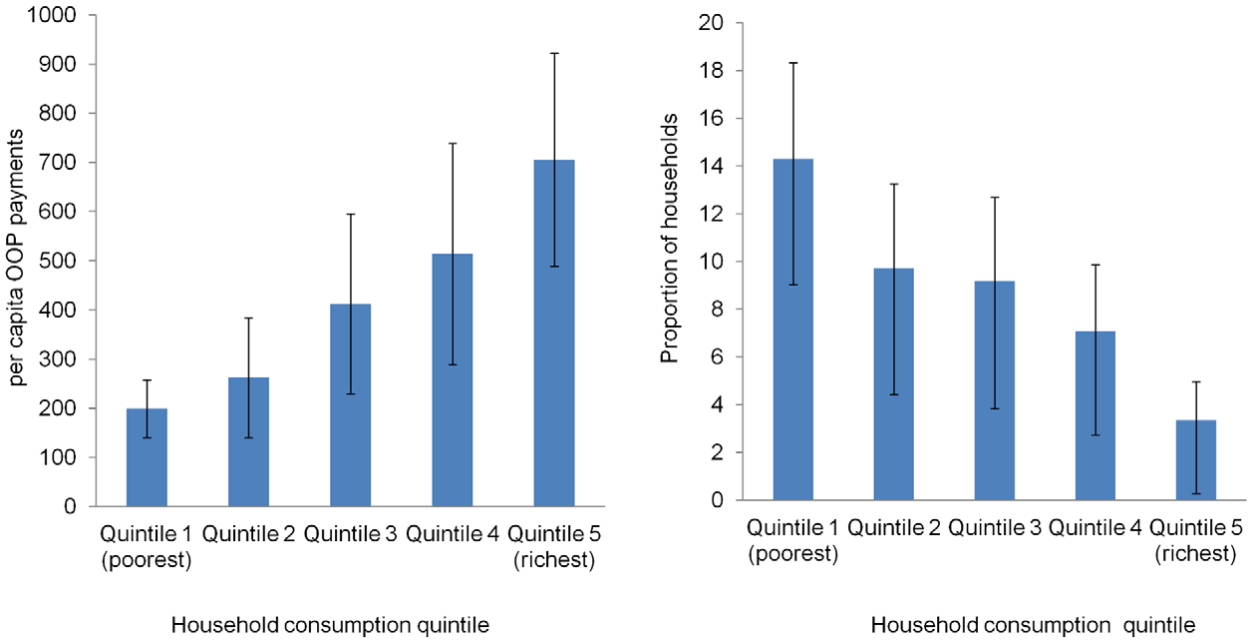
Association between household consumption quintile and per capita OOP payments and catastrophic expenditure.

The incidence of catastrophic healthcare expenditure by illness, care-seeking behavior and household level characteristics is presented in Table 1. Table 1 also shows household characteristics. Of the 1593 households sampled, average total monthly household consumption expenditure was TK 15749.0 (US $ 209.5) (95% CI 10064.3–23720.0), 91.2% (95% CI 88.5–93.2) had incurred positive health expenditure and the share of OOP payments was about 10.6% (95% CI 8.6–12.5) of total expenditure. On average, residents spent TK 138.0 (US$ 1.8) (95% CI 42.5–366.6) per month on health-related goods and services. During the past 30 days recall period, 1501 households (about 94%) had at least one illness episode. Of these, 1148 (71%) households had at least one chronic illness and the average number of illnesses was 2.8 per household (95% CI 2.6–2.9). Overall, nearly 9% of the households incurred catastrophic healthcare expenditure at a capacity to pay threshold of 40%. At a non-food expenditure threshold of 25% and 40%, the incidence of catastrophic expenditure was 9.8% and 17.6% respectively. Figure 1 shows the association between household consumption quintile and per capita OOP payments and proportion of households facing catastrophic health expenditure. There was a statistically significant trend towards higher OOP expenditure in wealthier households (p<0.01) but lower risk of catastrophic expenditure (p<0.01). The average total cost of illness per household, per capita monthly expenditure and the medical expenses intensity ratio (the ratio of these two variables) are presented in Table 2. The overall median cost of one illness episode and per capita monthly consumption expenditure was TK 242 and TK 3517 respectively and these costs differed significantly by consumption quintile (p-value for trend p<0.01). About 50% of residents spent an amount equivalent to at least 7% of monthly per capita consumption expenditure on one episode of illness.

**Table 1:** Incidence of catastrophic health expenditure by illness and household characteristics.

Variable	Frequency (n = 1593)	Frequency of catastrophic expenditure	Proportion (95% CI)	P-value
**Illness and care-seeking behavior**				
Care-seeking behavior				
Inpatient	65	44	68.5 (56.6–78.4)	<0.01
Outpatient public	253	23	9.0 (5.6–14.2)	
Outpatient private	385	35	9.3 (6.5–13.1)	
Outpatient public and private	105	14	16.9 (10.2–26.8)	
Self-medication/traditional healer	785	21	2.8 (1.6–4.8)	
Member with chronic disease				
Yes	1148	115	10.5 (8.3–13.3)	0.01
No	445	22	5.2 (3.1–8.5)	
**Household characteristics**				
Household member over 65 years				
Yes	136	16	11.0 (6.4–18.3)	0.4
No	1457	121	8.8 (7.0–11.1)	
Gender of household head				
Male	1447	124	8.9 (7.2–11.0)	0.9
Female	146	13	9.5 (4.2–19.9)	
Educational status of household head				
No education	258	36	15.2 (11.1–20.5)	<0.01
Primary	310	38	11.4 (7.8–16.6)	
Secondary	420	33	7.2 (5.0–10.2)	
Higher	605	30	5.9 (4.1–8.4)	
Household consumption quintile				
Quintile 1 (poorest)	319	47	14.3 (10.3–19.6)	<0.01
Quintile 2	319	30	9.7 (6.2–15.0)	
Quintile 3	318	30	9.2 (5.7–14.5)	
Quintile 4	319	20	7.1 (4.3–11.4)	
Quintile 5 (richest)	318	10	3.4 (1.7–6.4)	

All percentages and confidence intervals incorporate the effect of the probability sampling structure.

**Table 2:** Ratio between cost of illness per household and monthly expenditure per household member.

Characteristics	Median cost (TK) per illness episode	Median expenditure (TK) per household member	Median ratio of cost/income ratio
Consumption quintile			
Quintile 1 (poorest)	150	1785	0.08
Quintile 2	188	2623	0.07
Quintile 3	242	3481	0.07
Quintile 4	285	4997	0.06
Quintile 5 (richest)	467	7944	0.05
Total	242	3517	0.07
P-value for trend	P<0.01	P<0.01	P<0.01

### Determinants of OOP healthcare expenditure

Results of the double hurdle model are presented in Table 3. Because all subjects who received inpatient care incurred OOP payments, care-seeking behavior could not be included as a determinant of decision to spend, but was included in the second-stage equation. The participation and consumption decisions were not independent (χ^2^_(1)_ = 8.88; p<0.01), indicating a double hurdle model is appropriate for this data. Presence of chronic illness, household size, average illness per child and adult, care-seeking behavior, education level of the household head and household consumption quintile significantly affected the level of household OOP healthcare spending.

**Table 3:** Double–hurdle regression model of expenditure (total sample household data).

Variable	1st Stage			2nd stage		
	Participation (probit) equation			Expenditure (Tobit) equation		
	Coefficient	Standard error	p-value	Coefficient	Standard error	p-value
Constant	−0.7	0.34	0.04	4.81	0.16	<0.01
**Illness and care-seeking behavior**						
Average illness per child	0.17	0.07	0.02	0.1	0.02	<0.01
Average illness per adult	1.27	0.53	0.02	0.24	0.04	<0.01
Member with chronic disease						
Yes	0.49	0.13	<0.01	0.46	0.08	<0.01
No	0	NA		0	NA	
Care-seeking behavior						
Inpatient				3.17	0.13	<0.01
Outpatient public				0.78	0.07	<0.01
Outpatient private				1.21	0.08	<0.01
Outpatient both public and private				1.46	0.1	<0.01
Self-medication/traditional healer				0	NA	
**Household characteristics**						
Educational status of household head						
No education	−0.04	0.2	0.9	0.02	0.11	0.8
Primary	0.39	0.21	0.06	0.14	0.11	0.2
Secondary	0.2	0.13	0.1	0.19	0.09	0.03
Higher	0	NA		0	NA	
Age of household head (years)	0	0.01	0.7	0.01	0	<0.01
Household size	0.16	0.06	0.01			
Household consumption quintile						
Quintile 1 (poorest)				−0.62	0.12	<0.01
Quintile 2				−0.52	0.11	<0.01
Quintile 3				−0.29	0.1	0.01
Quintile 4				−0.24	0.11	0.03
Quintile 5 (richest)				0	NA	

### Determinants of catastrophic healthcare expenditure

Table 4 shows the results of the Poisson regression model of risk of catastrophic expenditure, defined as an expense in excess of 40% of the household capacity to pay (40% threshold) in the past 30 days. The average number of illnesses, both per child and per adult, significantly increased the relative risk of incurring catastrophic payments, by 1.12 times and 1.47 times for a single additional average illness in children and adults, respectively. The relative risk of catastrophic expenditure relative to households who used traditional healers or pharmacies only was higher for private than public outpatient facilities, and higher still for households who used both public and private outpatient facilities. Hospitalization was the biggest risk factor for catastrophic expenses. Households in the poorest quintile had more than four times the risk of catastrophic expenditure than the richest quintile and as the household head's education level declined the relative risk of catastrophic health expenditure increased.

**Table 4:** Multiple Poisson regression model for catastrophic expenditure.

Variable	Relative risk	95% confidence interval	p-value
**Illness and care-seeking behavior**			
Average illness per child	1.12	(1.03–1.23)	0.01
Average illness per adult	1.47	(1.13–1.93)	0.01
Care-seeking behavior			
Inpatient	28.36	(16.49–48.77)	<0.01
Outpatient public	2.93	(1.66–5.16)	<0.01
Outpatient private	4.38	(2.31–8.30)	<0.01
Outpatient public and private	7.03	(3.37–14.66)	<0.01
Self-medication/traditional healer	1.00	NA	
**Household characteristics**			
Educational status of household head			
No education	2.35	(1.25–4.41)	<0.01
Primary	1.62	(0.88–3.00)	0.1
Secondary	1.30	(0.74–2.27)	0.4
Higher	1.00	NA	
Household consumption quintile			
Quintile 1 (poorest)	3.76	(1.46–9.68)	<0.01
Quintile 2	2.55	(1.02–6.38)	0.01
Quintile 3	2.61	(1.22–5.54)	0.01
Quintile 4	2.25	(1.09–4.65)	0.01
Quintile 5 (richest)	1.00	NA	

## Discussion

This paper, based on a representative household survey in Rajshahi city, Bangladesh, is the first to consider illness, care-seeking behavior, demographics of the household head, and household economic characteristics as household-level predictors of OOP payments and catastrophic expenditure. It is also among the few examples of studies that have reported the incidence of catastrophic healthcare expenditure in Bangladesh [16], [17], and the first to estimate this incidence from a representative, probability-sampled survey.

This study found that sampled households, none of whom have any form of risk-pooling insurance, spend about 11% of their total household budget on healthcare, and nearly 9% of households experience financial catastrophe. At a 25% non-food expenditure threshold, the incidence of financial catastrophe was similar to another published study, at 18% [16]. The study demonstrated that the medical spending associated with an illnesses episode increased as household consumption expenditure increased, which is similar to another study in India [21]; however, we showed that despite this increase in spending, the risk of catastrophic expenditure decreased with household consumption expenditure. In addition to the common finding that household consumption quintile and receiving inpatient care are associated with financial catastrophe, this study showed the importance of the average number of illness episodes among children and adults, and the presence of chronic illness in a household as key determinants of high OOP payments and financial catastrophe. Higher levels of education in the household head were also protective against OOP spending and catastrophic expenditure, similar to other developing countries [8], [25].

This study revealed that the per capita monthly OOP health expenditure made by households was TK 138.0 (US$ 1.8), which is similar to national-level findings in Bangladesh [15]. The estimated proportion of catastrophic expenditure in our study is consistent with van Doorslaer et al [16] but contradicts the findings (1.2%) of Xu and colleagues [17], though our study supports their findings that poor households were less able to cope with any level of health payment than rich households [19], [40], [41]. The disagreement in proportions of catastrophic expenditure between our study and Xu et al is likely to be due to differences in data and measurement methods. Their study used the Bangladesh HIES, which mainly emphasized poverty assessment and was not designed to account for details of household illness and their treatment responses or costs. According to Xu and colleagues, the estimated proportion of households facing catastrophic expenditure in Bangladesh may be underestimated in the 1995 HIES survey due to missing information such as the absence of durable goods from the consumption calculation, and the very limited information collected on episode-of-illness level healthcare expenditure data, and care-seeking behavior. In their study, van Doorslaer and colleagues estimated the incidence of catastrophic expenditure based on total household consumption and non-food expenditure but they did not assess the incidence of catastrophic expenditure using household capacity to pay. In contrast, our study considered all household members who suffered any illness and their treatment response in the past 30 days, and then collapsed information into household level for analysis purposes. Therefore, our study offers more accurate information than the previous two studies conducted in Bangladesh, and also used a more detailed and accurate methodology for estimating the burden of OOP payments, with adjustments for household size and capacity to pay [42].

Consistent with other studies [19], [20], [22], [43]–[46], although the richest households reported more illness, spent more on health and utilized more private facilities compared to the poorest quintile, risk of financial catastrophe was higher in the poorest households, indicating that the burden of financial catastrophe falls disproportionately on the poor. The three key preconditions for catastrophic health expenditure are the presence of health services requiring payments, low capacity to pay, and lack of prepayment or health insurance options [17]. These conditions are all present in the poorest households in Bangladesh, and the high proportion of catastrophic expenses in the lowest quintiles points to the urgent need to remove one or all of these preconditions. For example, the OOP share dropped markedly following the introduction of health insurance in China [47], Vietnam [48], and India [49], and the introduction of even basic prepayment or health insurance systems in Bangladesh may have a similar effect on the poorest households.

Our analyses demonstrate a negative impact of average illness per child and adult, and presence of chronic illness in the household, on the household economy. These results are similar to the determinants of catastrophic expenditure in Burkina Faso and India [18], [20], such as lack of formal education, tuberculosis, diabetes, dementia, modern medical care, number of illness episodes among adults and chronic illness. In concordance with results from India [21], the level of OOP payments is higher among those who used inpatient care services and suffer from chronic illness. Moreover, the study also revealed the importance of the average number of illness episodes among children and adults, and larger household size as key factors responsible for high OOP payments. Chronic care for NCDs puts an enormous and continuous financial strain on household budgets. The costs of care of chronic NCDs often contribute to increased OOP payments, pushing households into impoverishment or below the poverty line [3], [50]. In such critical situations, only a strong risk pooling mechanism can prevent the poorest households from risk of financial catastrophe. Health insurance can have the dual function of protecting families against health shocks that increase healthcare needs, and against economic shocks that reduce their capacity to finance healthcare [51].

Type of health service used was also another important determinant of OOP payments and financial catastrophe, with intensity of OOP payments at public outpatient facilities lower than private outpatient facilities. These findings are similar to several studies from developing countries [8], [52] but at variance with a Nepalese study [25], [44]. Although public health facilities in Bangladesh are heavily subsidized by the Government [53], [54], the risk of incurring OOP expenditure as well as catastrophic spending remains high for users of these facilities. This suggests that subsidized programs may not be working properly among disadvantaged groups. One reason could be that unofficial fees in public facilities can significantly exceed the amounts expected in official payments, and fee exemptions are not always possible [53], suggesting that public facilities are not providing their expected financial protection in practice. Another possible reason is the need to purchase drugs and ancillary health services such as medicines or tests on the private market. This suggests the need for state-subsidized public clinics to provide more holistic and inclusive services. Finally, similarly to other studies [19], [43], those receiving inpatient care were at high risk of OOP expenditure and catastrophic spending. In the absence of a risk-pooling mechanism, all households face high risk of financial catastrophe from OOP payments for inpatient care.

The research protocol and sampling process in our study was designed to avoid any biases in the results, but our study has several limitations. We examined only urban households in one metropolitan area of the country, so the results cannot necessarily be generalized to the whole country. However, the representative nature of the sample means that the results may be applicable to other cities, and thus the study may reflect the reality of health market participation for a large proportion of the Bangladeshi population. Inpatient service use is infrequent (4%) and a much larger sample is required to explore the role of chronic vs. acute illness in hospitalization and costs. Such an analysis might better describe the role of preventable hospital admissions in catastrophic spending. Consumption and expenditure were self-reported and prone to error, although estimates were confirmed by other household members or aged persons in the community. For example, female interviewees frequently over- or under-estimated the cost of bicycles, sewing machines and cars, but we minimized the bias by asking another household member or an older member of the household.

This study identifies determinants of high medical expenditure and financial catastrophe: illness either in children or in adults, chronic illness, receiving inpatient care, poorer economic status and lower education level of the household head. The chronic care of NCDs requires long-term routine clinic visits, testing, and medications, reducing households’ flexibility to respond to the cost of unexpected hospitalization or other illness episodes. It is clear that immediate action is necessary to reduce levels of catastrophic health expenditure by reducing the burden of OOP payments in Bangladesh, which can be achieved by:

- Implementing compulsory health insurance for salaried workers in both public and private sectors, and voluntary memberships for dependents, farmers and self-employed persons, similarly to programs in Vietnam that have been shown to reduce OOP payments to lower levels than observed in this study [55]
- Improving routine management of NCDs, to reduce the cost of chronic disease management, and incorporating chronic disease management into public services and health financing initiatives, to ensure that this expenditure is included in risk-pooling and welfare initiatives and the high OOP payments associated with chronic illness that were identified in this study can be ameliorated by better and more equitable management, prevention and treatment
- Incorporating ancillary services into basic care packages in public facilities, so that users are not required to pay significant OOP expenses for essential pharmaceutical or other ancillary services which are supposed to be almost free, but which our study found were still associated with high OOP payments and catastrophic expenditure risk

If necessary reforms are implemented, especially those targeted at the poorest members of Bangladeshi society, significant reductions in the burden of OOP payments can be made, with consequent improvements in the health of the population.
